# Evaluation of Various Methods of Liver Measurement in Comparison to Volumetric Segmentation Based on Computed Tomography

**DOI:** 10.3390/jcm13133634

**Published:** 2024-06-21

**Authors:** Maciej Cebula, Angelika Biernacka, Oskar Bożek, Bartosz Kokoszka, Sylwia Kazibut, Anna Kujszczyk, Monika Kulig-Kulesza, Sandra Modlińska, Jakub Kufel, Michał Azierski, Filip Szydło, Mateusz Winder, Joanna Pilch-Kowalczyk, Katarzyna Gruszczyńska

**Affiliations:** 1 Individual Medical Practice, 40-754 Katowice, Poland; 2 Department of Radiodiagnostics and Invasive Radiology, University Clinical Center Prof. Kornel Gibiński of the Medical University of Silesia in Katowice, 40-752 Katowice, Poland; 3 Department of Radiodiagnostics, Invasive Radiology and Nuclear Medicine, Faculty of Medical Sciences, Medical University of Silesia, 40-752 Katowice, Poland; 4 Department of Radiology and Nuclear Medicine, Faculty of Medical Sciences, Medical University of Silesia, 40-752 Katowice, Poland; 5 Department of Radiology and Radiodiagnostics in Zabrze, Medical University of Silesia, 41-800 Katowice, Poland; 6 Students’ Scientific Association of MedTech, Medical University of Silesia, 40-055 Katowice, Poland; 7 Students’ Scientific Association of Computer Analysis and Artificial Intelligence, Department of Radiology and Nuclear Medicine, Medical University of Silesia, 40-752 Katowice, Poland

**Keywords:** liver, volumetry, computed tomography

## Abstract

**Background**: A reliable assessment of liver volume, necessary before transplantation, remains a challenge. Our work aimed to assess the differences in the evaluation and measurements of the liver between independent observers and compare different formulas calculating its volume in relation to volumetric segmentation. **Methods**: Eight researchers measured standard liver dimensions based on 105 abdominal computed tomography (CT) scans. Based on the results obtained, the volume of the liver was calculated using twelve different methods. An independent observer performed a volumetric segmentation of the livers based on the same CT examinations. **Results**: Significant differences were found between the formulas and in relation to volumetric segmentation, with the closest results obtained for the Heinemann et al. method. The measurements of individual observers differed significantly from one another. The observers also rated different numbers of livers as enlarged. **Conclusions**: Due to significant differences, despite its time-consuming nature, the use of volumetric liver segmentation in the daily assessment of liver volume seems to be the most accurate method.

## 1. Introduction

The assessment of liver size based on physical examination is disadvantageous, with a significant error possible due to considerable dependence on the experience of the examiner [1]. Therefore, a more reliable quantitative assessment of liver enlargement is possible in imaging studies, especially computed tomography (CT) and magnetic resonance (MRI). Volume estimation based on CT measurements is superior to that based on ultrasound, but its margin of error is still evident [2]. Three-dimensional imaging methods seem to be more accurate in this regard than two-dimensional ones. Currently, the need to move from two-dimensional to three-dimensional analysis is discussed more and more often, especially in the context of liver surgery. Although 3D segmentation technologies have been implemented for over a decade, traditional methods are still widely used in clinical practice. Our study aims to compare these older methods with modern techniques to better understand their limitations and provide insights into their comparison with current advanced techniques [3]. Currently, work has already begun on the use of the convolutional neural network to automate liver segmentation across different imaging modalities. While 3D liver segmentation is considered highly accurate, it is not without errors. Future studies could include post-surgical liver weighing and volume measurement as a more reliable reference standard [4,5]. The repeatability and reproducibility of liver measurements is a significant problem, especially in the case of manual measurements [6]. As liver volume estimation is mandatory prior to a living donor transplantation or partial resection, the mismatch between imaging and intraoperative findings is still a reported issue [7,8]. The tumor volume is a known confounding factor. The future remnant to total liver size assessment is faster when the total volume of the liver can be calculated from biometric data. The estimated total liver volume based on biometrics has been proposed particularly in order to size liver grafts in transplants [9]. Over the years, the number of formulas used to calculate the volume of the liver has increased, which has made it difficult to choose the best one. The use of such formulas may lead to over- or underestimation, which again may lead to an error in the assessment of potential liver failure after resection or transplantation. The number of publications comparing the proposed methods with volumetry remains scarce.

Our work aimed to assess (1) if the measurement of one of the dimensions of the liver allows for a reliable estimation of its volume, (2) if we can reliably estimate the liver volume based on linear liver measurements and the patient’s physiological data, (3) if hepatomegaly can be reliably qualified without taking measurements, and (4) if liver measurements based on simple instructions vary significantly between observers. In search of answers to the above questions, we relied on the method of computed tomography.

## 2. Materials and Methods

A total of 105 CT examinations (performed between October 2021 and March 2022) of the abdominal cavity have been selected and anonymized by an independent observer with five years of CT evaluation experience from the archives of the Department of Radiodiagnostics and Interventional Radiology, University Clinical Center prof. K. Gibiński of the Medical University of Silesia in Katowice, Poland. The CT images that were included in the liver had to have visible hepatic veins and no focal lesions. All analyzed liver computed tomography examinations were performed on a SIEMENS SOMATOM Definition Edge device using the three-phase liver protocol or the classic abdominal protocol. The inclusion criteria included an examination performed in at least two phases after administering intravenous contrast agent, no focal lesions, no signs of significant fibrosis, no previous surgeries on the liver, and no signs of cholestasis. All observers measured the liver using all methods. To increase accuracy, it would be beneficial to have more than one radiologist perform 3D liver segmentation. The sex, age, weight, and height of participants were noted, and their BMI and BSA were calculated. The selected group consisted of 27 men and 78 women aged 57.13 ± 16.44 years (56.74 ± 16.66 years and 57.27 ± 16.47 years, respectively) with a BMI of 25.40 ± 5.51 kg/m^2^ (24.88 ± 4.40 kg/m^2^ and 25.59 ± 5.87 kg/m^2^, respectively).

In the next step, a group of 8 observers, composed of 2 radiology specialists, 5 radiology residents, and 1 medical physicist with 4.13 ± 1.46 years of experience in CT evaluation, was tasked with assessing the following parameters using all methods: whether, in their opinion, the liver is enlarged; the maximum anteroposterior (AP), craniocaudal (CC), and left–right (SD) dimensions of the liver; and the AP and CC dimensions in the midclavicular line. The liver volume was calculated according to Muggli, David et al., Chan et al., Fu-gui et al., Urata et al., Hashimoto et al., Yuan et al., Poovathumkadavil, Vauthney et al. (BSA and Weight-based), Lin, Yu et al. and Heinemann et al. [10,11,12,13,14,15,16,17,18,19,20,21]. 

Below, we present the methods used in this work to calculate the volume of the liver based on the measurements made by the observers:

Muggli, David et al.:

Liver volume (mL) = ap (cm) × cor (cm) × cc (cm) × 0.31(1)

Chan et al.:

Liver volume (mL) = 302.34 + (218 + (age × 12.3) + sex × 51) × 0.859(2)

where age was counted as 1 for those <40, 2 if 41–60, and 3 if >60 years old and sex 1 for male and 0 for female.

Fu-Gui et al.:

Liver volume (mL) = 11.508 × body weight (kg) + 334.024(3)

Urata et al.:

Liver volume (mL) = 2.223 × weight (kg) × 0.426 × height (cm)^0.682^ (4)

Hashimoto et al.:

Liver volume (mL) = −404.8 + 961.3 × weight (kg)(5)

Yuan et al.:

Liver volume (mL) = 949.7 × body surface area (m^2^) − 48.3 × age − 247.4 (6)

where age was counted as 1 for those <40, 2 if 41–60, and 3 if >60 years old.

Poovathumkadavil:

Liver volume (mL) = 12.26 × body weight (kg) + 555.65(7)

Vauthney et al.’s BSA-based method:

Liver volume (mL)= −794.41 + 1267.28 × body surface area (m^2^) (8)

Vauthney et al.’s weight-based method:

Liver volume (mL) = 191.80 + 18.51 × weight (kg)(9)

Lin:

Liver volume (mL) = 13 × height (cm) + 12 × weight (kg) − 1530(10)

Yu et al.:

Liver volume (mL) = 21.585 × height (cm)^0.732^ × weight (kg)^0.225^ (11)

Heinemann et al.:

Liver volume (mL) = 1072.8 × body surface area (m^2^) − 345.7 (12)

The Mosteller method has been used for body surface area calculations [21]:

BSA (m^2^) = height (cm) × weight (kg)/3600)^½^ (13)

The observers rated the studies independently of each other using diagnostic software. Measurements were made in millimeters, with an accuracy of one decimal place. 

**Figure 1:**
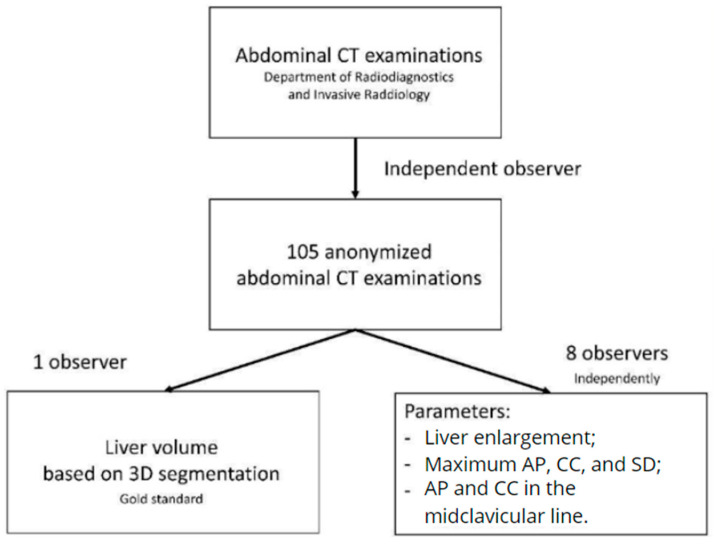
Diagram presenting the workflow at the stage of data collection and preparation.

At the same time, another observer, a radiology specialist with seven years of experience in CT analysis, performed 3D liver segmentations for the above examinations. The segmentation was performed with the liver analysis module of the *syngo*.via (Siemens Healthineers: Erlangen, Germany. *syngo*.via, version VB30A), according to the manufacturer’s instructions. The segmentation was conducted according to the manufacturer’s instructions; the liver outline was automatically detected, followed by a semi-automatic segmentation of hepatic veins and the portal vein after setting seed points. Axial images of the portal venous phase were used for obtaining the liver volume. The liver outline was segmented automatically, based on a hierarchical, learning-based approach. After the detection of the liver outline, hepatic veins and the portal vein were segmented semiautomatically. After setting seed points to the hepato-caval confluence and the main stem of the portal vein, the system automatically segments the hepatic veins and portal vein. The volumes of the intrahepatic vessels were included in the CT volume. Surrounding extrahepatic vessels, extrahepatic bile ducts, and the gallbladder were excluded. The liver volume obtained as a result of segmentation was adopted in the study as the gold standard against which other parameters and calculated volumes were compared [22,23,24]. For a better understanding, the workflow has been presented in Figure 1 and the data of the test group has been presented in Table 1.

The results were gathered and analyzed using Statistica 13.3 (TIBCO Software Inc.: Palo Alto, CA, USA (2017)). Statistica (data analysis software system) version 13. The normality of distribution was checked with a Kolmogorov–Smirnov test. Due to the abnormal distribution of most of the analyzed quantitative parameters, Mann–Whitney and Kruskal–Wallis tests were implemented. A Spearman test was used for the correlation strength analysis. The qualitative variables were analyzed with the Pearson test. Using stepwise regression, an attempt was made to build a model calculating the liver volume based on the collected data. As the value of statistical significance, a *p* of 0.05 was adopted.

Due to the retrospective nature of the work and the anonymization of the patients’ data, a full ethical review, approval, and patient consent were waived for this study by the Ethical Committee of Medical University of Silesia in Katowice, Poland.

## 3. Results

### 3.1. Liver Volume in Relation to Other Variables

The analysis of single measurement and parameter correlations with the segmented liver volume showed that there was a significant correlation (*p* < 0.001) for all of them; their correlation coefficients (r) and determination coefficients (r^2^) are presented in Table 2.

**Figure 2:**
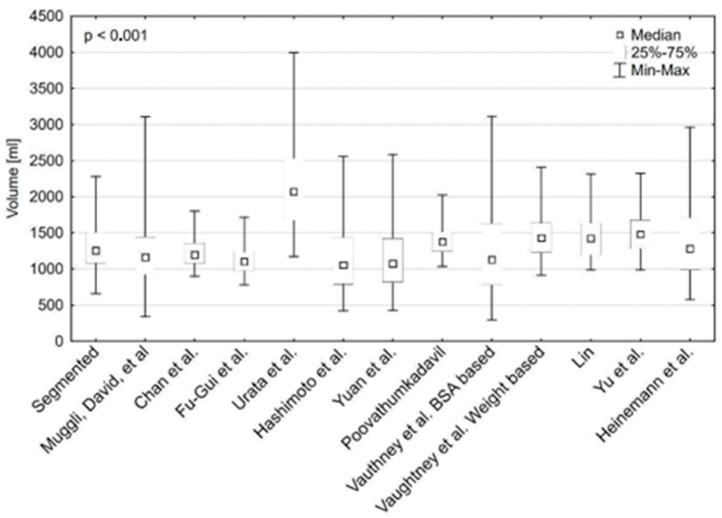
Difference between volumes calculated with each method and segmented liver volumes [11,12,13,14,15,16,17,18,19,20,21].

The difference between the calculated and segmented liver volumes is significant (*p* < 0.001) and presented in Figure 2. The results of multiple two-sided comparisons are attached as Supplementary Material Table S1.

An attempt to build an optimized model based on the acquired data was performed with stepwise regression (both stepwise and progressive). Considering only the measurements made by observers, a viable model was calculated with a corrected r^2^ = 0.749 based on maximum AP, CC, and SD measurements and the CC in the midclavicular line. With the addition of the rest of the acquired quantitative variables, a viable model with a corrected r^2^ of 0.824 and *p* < 0.001 was obtained, based on the measurements above, with the addition of age and weight. 

### 3.2. Differences between the Observers

There was a significant difference observed for all of the measurements made by the observers, but the calculated volumes differed only for the Muggli, David et al. method. The *p* values are shown in Table 3.

The difference between the observers in terms of the number of livers marked as enlarged was also significant, *p* < 0.001; the results are presented in Table 4.

## 4. Discussion

As can be seen in our results, the repeatability of liver measurements between independent observers is a factor that can significantly affect the assessment of a liver’s volume. The obtained results confirm the previous reports that it is impossible to reliably estimate liver volume based on a single measurement. To minimize inter-operator variability, future studies should include multiple operators for 3D measurements. In our study, we used a single operator to ensure consistency; however, we recommend considering inter-operator variability in further analyses [7]. Liver-volume-calculating formulas based on biological data, such as patients’ weight and height, seem not to be significantly affected by this problem, as their results were comparable to each other. The observers’ years of experience in CT evaluation was not a significant factor affecting the measurements. As reported before, only minimal training is needed to reliably make these measurements [25].

Of all analyzed methods, only Heinemann et al. offered results comparable to volumetric segmentation. The obtained results are consistent with the reports to date on the significant differences between the analyzed formulas used for assessing the liver volume [10,26].

The evaluation of the presence of hepatomegaly without measurements is not reliable due to the significant differences in this assessment.

So far, many authors have presented various solutions to the problem of liver volume measurement, but each of the proposed methods is burdened with some error [10,26]. Currently, there is no consensus on which calculation method based on patient’s physiological data is better [27]. Even automated liver segmentation, used as the gold standard in this work, is affected by factors such as scan phase and slice thickness [8,28]. The CT and MRI methods seem to correlate strongly; thus, the use of only magnetic resonance imaging to assess liver volume does not appear to be economically viable [29]. The implementation of automatic methods of liver segmentation allowed for a significant reduction in the process time compared to semi-automatic and manual methods, which should significantly facilitate this type of measurement in everyday practice [30].

To average out possible uncertainties due to individual biases and/or experience levels, the measurements were performed by a group of specialists with a wide range of experience years. Another limitation of the method is the measurement of examinations obtained from the same CT scanner. The last limitation of the method was the small number of studies on which the analysis was based.

## 5. Conclusions

Our study concluded that a single liver measurement does not allow for reliable volume estimation. Most methods based on linear measurements and patient physiological data do not provide accurate liver volume estimates. Hepatomegaly cannot be reliably assessed without measurements. Liver measurements based on simple instructions vary significantly between observers. The use of volumetric liver segmentation in daily volume assessments appears to be the most accurate and time-optimized method. Implementing 3D segmentation techniques in clinical practice can significantly improve the accuracy of preoperative assessments and patient outcomes. This study highlights the significant discrepancies between various liver volume estimation methods and underscores the importance of transitioning to more reliable and accurate 3D segmentation techniques in clinical practice. Our results confirm previous reports of significant differences between the analyzed formulas in liver volume assessments. The significant differences in measurements between observers highlight the need for more standardized and precise methods, such as 3D segmentation. Future research should focus on further improving automatic segmentation methods and their implementation in daily clinical practice to minimize errors and improve preoperative outcomes. By adopting volumetric liver segmentation, clinicians can achieve more precise assessments, potentially improving preoperative planning and patient outcomes.

## Figures and Tables

**Table 1:** Data of the analyzed group.

Variable	Value
Gender [n (%)]	
Females	78 (74.29)
Males	27 (25.71)
Age [years]	57.13 ± 16.44
Height [cm]	164.64 ± 9.38
Weight [kg]	69.15 ± 17.16
BMI [kg/m^2^]	25.40 ± 5.51
BSA [m^2^] ^1^	1.59 ± 0.46
Maximum measurement [mm]	
AP	152.34 ± 20.66
CC	142.36 ± 24.48
SD	178.12 ± 31.34
Midclavicular measurement [mm]	
AP	140.48 ± 23.05
CC	115.57 ± 22.85
Liver marked as enlarged ^2^	17.00 ± 9.72
Calculated liver volume [mL]:	
Muggli, David et al. [11]	1216.96 ± 412.70
Chan et al. [12]	1232.22 ± 188.13
Fu-Gui et al. [13]	1129.86 ± 197.48
Urata et al. [14]	2137.31 ± 582.78
Hashimoto et al. [15]	1126.60 ± 437.89
Yuan et al. [16]	1155.74 ± 437.18
Poovathumkadavil [17]	1403.49 ± 210.39
Vaughtney et al. [18]—BSA-based method	1224.43 ± 577.27
Vaughtney et al. [18]—weight-based method	1471.85 ± 317.64
Lin [19]	1440.13 ± 289.35
Yu et al. [20]	1504.83 ± 282.35
Heinemann et al. [21]	1363.32 ± 488.68
Segmented liver volume [mL]	1345.32 ± 350.57

^1^ Calculated with the Mosteller method. ^2^ Presented as mean and standard deviation of the number of livers marked as enlarged by each observer.

**Table 2:** Correlation and determination coefficients of single measurements and parameters with the segmented liver volume.

Variable	Correlation Coefficient (r)	Determination Coefficient (r^2^)
Measurement		
Maximum AP	0.680	0.462
Maximum CC	0.581	0.338
Maximum SD	0.396	0.157
Midclavicular AP	0.570	0.325
Midclavicular CC	0.477	0.227
Parameter		
Age	−0.229	0.052
Height	0.468	0.219
Weight	0.664	0.441
BMI	0.515	0.266
BSA	0.672	0.452

**Table 3:** Differences between observers in terms of liver measurements and calculated liver volumes.

Measurement	Kruskal–Wallis Test Result (*p*)
Maximum AP	0.001
Maximum CC	<0.001
Maximum SD	<0.001
Midclavicular AP	<0.001
Midclavicular CC	<0.001
Calculated liver volume [mL]:	
Muggli, David et al. [11]	<0.001
Chan et al. [12]	1.0
Fu-Gui et al. [13]	1.0
Urata et al. [14]	1.0
Hashimoto et al. [15]	1.0
Yuan et al. [16]	1.0
Poovathumkadavil [17]	1.0
Vaughtney et al. [18]—BSA-based method	1.0
Vaughtney et al. [18]—weight-based method	1.0
Lin [19]	1.0
Yu et al. [20]	1.0
Heinemann et al. [21]	1.0

**Table 4:** Difference between the observers in terms of the number of livers marked as enlarged.

Observer	Enlarged Livers	Not Enlarged Livers
I	14	91
II	31	74
III	7	98
IV	10	95
V	5	100
VI	19	86
VII	21	84
VIII	29	76

## Data Availability

Data are available from the corresponding author on request.

## Supplementary Materials

The following supporting information can be downloaded at: https://www.mdpi.com/article/10.3390/jcm13133634/s1, Table S1. The results of two-sided multiple comparisons as result of Kruskal-Wallis post-hoc tests, presented as *p* value. Significant differences are marked in red.

## References

[1] Joshi R., Singh A., Jajoo N., Pai M., Kalantri S.P. (2004). Accuracy and reliability of palpation and percussion for detecting hepatomegaly: A rural hospital-based study. Indian J. Gastroenterol..

[2] Seppelt D., Kromrey M.L., Ittermann T., Kolb C., Haubold A., Kampfrath N., Fedders D., Heiss P., Hoberück S., Hoffmann R.T. (2022). Reliability and accuracy of straightforward measurements for liver volume determination in ultrasound and computed tomography compared to real volumetry. Sci. Rep..

[3] Radtke A., Sotiropoulos G.C., Nadalin S., Molmenti E.P., Schroeder T., Saner F.H., Sgourakis G., Cicinnati V.R., Valentin-Gamazo C., E Broelsch C. (2008). Preoperative volume prediction in adult live donor liver transplantation: 3-D CT volumetry approach to prevent miscalculations. Eur. J. Med. Res..

[4] Fang C., An J., Bruno A., Cai X., Fan J., Fujimoto J., Golfieri R., Hao X., Jiang H., Jiao L.R. (2020). Consensus recommendations of three-dimensional visualization for diagnosis and management of liver diseases. Hepatol. Int..

[5] Wang K., Mamidipalli A., Retson T., Bahrami N., Hasenstab K., Blansit K., Bass E., Delgado T., Cunha G., Middleton M.S. (2019). Automated CT and MRI liver segmentation and biometry using a generalized convolutional neural network. Radiol. Artif. Intell..

[6] Kavur A.E., Gezer N.S., Baris M., Sahin Y., Ozkan S., Baydar B., Yuksel U., Kilikcier C., Olut S., Akar G.B. (2020). Comparison of semi-automatic and deep learning-based automatic methods for liver segmentation in living liver transplant donors. Diagn. Interv. Radiol..

[7] Hermoye L., Laamari-Azjal I., Cao Z., Annet L., Lerut J., Dawant B.M., Van Beers B.E. (2005). Liver segmentation in living liver transplant donors: Comparison of semiautomatic and manual methods. Radiology.

[8] Radtke A., Sotiropoulos G.C., Nadalin S., Molmenti E.P., Schroeder T., Lang H., Saner F., Valentin-Gamazo C., Frilling A., Schenk A. (2007). Preoperative volume prediction in adult living donor liver transplantation: How much can we rely on it? Essen experience based on virtual three-dimensional computed tomography-volume assessment. Am. J. Transplant..

[9] Mortelé K.J., Cantisani V., Troisi R., de Hemptinne B., Silverman S.G. (2003). Preoperative liver donor evaluation: Imaging and pitfalls. Liver Transplant..

[10] Pomposelli J.J., Tongyoo A., Wald C., Pomfret E.A. (2012). Variability of standard liver volume estimation versus software-assisted total liver volume measurement. Liver Transplant..

[11] Muggli D., Müller M., Karlo C., Fornaro J., Marincek B., Frauenfelder T., Davda S., Kowa X.-Y., Aziz Z., Ellis S. (2009). A simple method to approximate liver size on cross-sectional images using living liver models. Clin. Radiol..

[12] Chan S.C., Liu C.L., Lo C.M., Lam B.K., Lee E.W., Wong Y., Fan S.T. (2006). Estimating liver weight of adults by body weight and gender. World J. Gastroenterol..

[13] Fu-Gui L., Lu-Nan Y., Bo L., Yong Z., Tian-Fu W., Ming-Qing X., Wen-Tao W., Zhe-Yu C. (2009). Estimation of standard liver volume in Chinese adult living donors. Transplant. Proc..

[14] Urata K., Kawasaki S., Matsunami H., Hashikura Y., Ikegami T., Ishizone S., Momose Y., Komiyama A., Makuuchi M. (1995). Calculation of child and adult standard liver volume for liver transplantation. Hepatology.

[15] Hashimoto T., Sugawara Y., Tamura S., Hasegawa K., Kishi Y., Kokudo N., Makuuchi M. (2006). Estimation of standard liver volume in Japanese living liver donors. J. Gastroenterol. Hepatol..

[16] Yuan D., Lu T., Wei Y.G., Li B., Yan L., Zeng Y., Wen T., Zhao J. (2008). Estimation of standard liver volume for liver transplantation in the Chinese population. Transplant. Proc..

[17] Poovathumkadavil A., Leung K.F., Al Ghamdi H.M., Othman I.H., Meshikhes A.W. (2010). Standard formula for liver volume in Middle Eastern Arabic adults. Transplant. Proc..

[18] Vauthey J.N., Abdalla E.K., Doherty D.A., Gertsch P., Fenstermacher M.J., Loyer E.M., Lerut J., Materne R., Wang X., Encarnacion A. (2002). Body surface area and body weight predict total liver volume in Western adults. Liver Transpl..

[19] Lin X.Z., Sun Y.N., Liu Y.H., Sheu B.S., Cheng B.N., Chen C.Y., Tsai H.M., Shen C.L. (1998). Liver volume in patients with or without chronic liver diseases. Hepatogastroenterol..

[20] Yu H., You H., Lee H., Jin Z., Moon J., Cho B. (2004). Estimation of standard liver volume for liver transplantation in the Korean population. Liver Transplantation.

[21] Heinemann A., Wischhusen F., Puschel K., Rogiers X. (1999). Standard liver volume in the Caucasian population. Liver Transpl. Surg..

[22] Mosteller R.D. (1987). Simplified calculation of body-surface area. N. Engl. J. Med..

[23] Lim S.J., Jeong Y.Y., Ho Y.S. (2006). Automatic liver segmentation for volume measurement in CT Images. J. Vis. Commun. Image Represent..

[24] Tanpowpong N., Yimpraphan S., Vajragupta L., Sirijindakul B., Nunthasoot B. (2007). Accuracy of liver volume measurement using multidetector computed tomography. Asian Biomed..

[25] Masperi A., Cubadda V., Bombelli L., Labruna R., Bagnardi V., Fodor C.I., Pagan E., Bonomo G., Orsi F. (2021). Intra-inter-observer repeatability in liver computed tomography volumetry in patients undergoing radioembolization simulation. Abdom. Radiol..

[26] Olthof P.B., van Dam R., Jovine E., Campos R.R., de Santibañes E., Oldhafer K., Malago M., Abdalla E.K., Schadde E. (2019). Accuracy of estimated total liver volume formulas before liver resection. Surgery.

[27] Lim M., Tan C., Cai J., Zheng J., Kow A. (2014). CT volumetry of the liver: Where does it stand in clinical practice. Clin. Radiol..

[28] Hori M., Suzuki K., Epstein M.L., Baron R.L. (2011). Computed tomography liver volumetry using 3-dimensional image data in living donor liver transplantation: Effects of the slice thickness on the volume calculation. Liver Transpl..

[29] Karlo C., Reiner C.S., Stolzmann P., Breitenstein S., Marincek B., Weishaupt D., Frauenfelder T. (2010). CT- and MRI-based volumetry of resected liver specimen: Comparison to intraoperative volume and weight measurements and calculation of conversion factors. Eur. J. Radiol..

[30] Perandini S., Faccioli N., Inama M., Mucelli R.P. (2011). Freehand liver volumetry by using an electromagnetic pen tablet: Accuracy, precision, and rapidity. J. Digit. Imaging.

