# Supplementary material for: Evaluation of Various Methods of Liver Measurement in Comparison to Volumetric Segmentation Based on Computed Tomography
Source: J Clin Med. 2024 Jun 21;13(13):3634. doi: 10.3390/jcm13133634 (PMC11242708; doi:10.3390/jcm13133634)
Supplement: Supplementary file 1 [file jcm-13-03634-s001.zip › jcm-3038317-supplementary.pdf]

Table S1. The results of two-sided multiple comparisons as result of Kruskal-Wallis post-hoc tests, presented as p value. Significant differences are marked in red.

Variable: Volume	Kruskal-Wallis test: p =0,000												
	Segmented	Muggli, David, et al	Chan et al.	Fu-Gui et al.	Urata et al.	Hashimoto et al.	Yuan et al.	Poovathumkadavil	Vauthney et al. BSA based	Vaughtney et al. Weight based	Lin	Yu et al.	Heinemann et al.
Segmented		< 0.001	0.001	< 0.001	< 0.001	< 0.001	< 0.001	< 0.001	< 0.001	< 0.001	< 0.001	< 0.001	1.000
Muggli, David, et al	< 0.001		1.000	0.003	< 0.001	0.031	0.757	< 0.001	1.000	< 0.001	< 0.001	< 0.001	< 0.001
Chan et al.	0.001	1.000		< 0.001	< 0.001	0.001	0.039	< 0.001	1.000	< 0.001	< 0.001	< 0.001	0.138
Fu-Gui et al.	< 0.001	0.003	< 0.001		< 0.001	1.000	1.000	< 0.001	0.045	< 0.001	< 0.001	< 0.001	< 0.001
Urata et al.	< 0.001	< 0.001	< 0.001	< 0.001		< 0.001	< 0.001	< 0.001	< 0.001	< 0.001	< 0.001	< 0.001	< 0.001
Hashimoto et al.	< 0.001	0.031	0.001	1.000	< 0.001		1.000	< 0.001	0.278	< 0.001	< 0.001	< 0.001	< 0.001
Yuan et al.	< 0.001	0.757	0.039	1.000	< 0.001	1.000		< 0.001	1.000	< 0.001	< 0.001	< 0.001	< 0.001
Poovathumkadavil	< 0.001	< 0.001	< 0.001	< 0.001	< 0.001	< 0.001	< 0.001		< 0.001	1.000	1.000	0.086	< 0.001
Vauthney et al. BSA based	< 0.001	1.000	1.000	0.045	< 0.001	0.278	1.000	< 0.001		< 0.001	< 0.001	< 0.001	< 0.001
Vaughtney et al. Weight based	< 0.001	< 0.001	< 0.001	< 0.001	< 0.001	< 0.001	< 0.001	1.000	< 0.001		1.000	1.000	< 0.001
Lin	< 0.001	< 0.001	< 0.001	< 0.001	< 0.001	< 0.001	< 0.001	1.000	< 0.001	1.000		0.368	< 0.001
Yu et al.	< 0.001	< 0.001	< 0.001	< 0.001	< 0.001	< 0.001	< 0.001	0.086	< 0.001	1.000	0.368		< 0.001
Heinemann et al.	1.000	< 0.001	0.139	< 0.001	< 0.001	< 0.001	< 0.001	< 0.001	< 0.001	< 0.001	< 0.001	< 0.001	
